# Supplementary material for: Using an integrated knowledge translation approach to inform a pilot feasibility randomized controlled trial on peer support for individuals with traumatic brain injury: A qualitative descriptive study
Source: PLoS One. 2021 Aug 24;16(8):e0256650. doi: 10.1371/journal.pone.0256650 (PMC8384186; doi:10.1371/journal.pone.0256650)
Supplement: S1 File — (PDF) [file pone.0256650.s001.pdf]

Interview Guide – Phase 1
A Randomized controlled feasibility trial on the Ontario Brain Injury Association Peer Network

Pre-interview Routine
- Consent Form
- Honorarium
- Demographic/Descriptive Information

Demographic/Descriptive Information

	Mentor	*Partner*	*Caregiver*	*OBIA staff*	*Researchers*
**Participant type**					
**Age**				*N/A*	*N/A*
**Moderate or severe TBI**			*NA*	*NA*	*NA*
**Number of years with TBI (i.e. time since injury)**			*NA*	*NA*	*NA*
**Number of years of experience with OBIA Peer Support Program**		*NA*	*NA*	*NA*	*NA*
**Relationship to the individual with TBI**	*NA*	*NA*		*NA*	*NA*
**Working Status/Job Title, Number of years of experience**					

*In our discussion today, we will use the term peer support, Mentor, or Partner. These are terms that the Ontario Brain Injury Association (OBIA) Peer Support program uses. We are defining **peer support** as a process in which an individual whose life experience helps another person currently coping with a similar event or issue and is in need of support from a knowledgeable friend. **Mentors** are individuals with a brain injury who provide ideas to help problem solve, knowledge, and/or guidance to a fellow person with a brain injury. Finally, **Partners** are individuals who have received ideas to problem solve, knowledge, and/or guidance from a Mentor (i.e., peer with a brain injury). Partners may be the caregivers of individuals with a brain injury as well. How do you feel about these words? Are you comfortable for us to use the terms peer support, Mentor, and Partner today?*

Question	Stakeholder Group
**Have you participated in the OBIA Peer Support Program?**<br><br>If YES…go to description of the TOPS research project.<br>If NO…then review the description of program **and** the description of the TOPS research project.	Mentor<br>Partner<br>Caregiver
**Are you familiar with the OBIA Peer Support Program?**<br><br>If YES…go to description of the TOPS research project.<br>If NO…then review the description of program **and** the description of the TOPS research project.	OBIA Staff<br>Researchers

Description of the OBIA Peer Support Program

*The Ontario Brain Injury Association (OBIA) Peer Support Program matches volunteer Mentors and Partners (so, individuals **receiving** the peer support) for a series of one-to-one interactions that focus on the discussion and resolution of problems or issues (i.e., problem solving) related to one or several key topic areas including family and friends, resources, life changes or challenges, (health care) professionals, social/recreational activities, work/employment/training/volunteering, the brain injury itself, emotions or feelings, and other issues. Once the match is established, the Mentor and Partner communicate with one another primarily by telephone. Most telephone calls last between 20 and 40 minutes and are held weekly. For some partnerships, communication by email or video conferencing is used. Matches typically last for one year.*

Description of the TOPS Research Project

*For the current research project, we would like to study the impact of the OBIA Peer Support Program. To do this, we will conduct a 6-month study where we randomly assign Partners to one of three groups – participating in the OBIA Peer Support program two times per week, participating in the OBIA Peer Support program once per week, or being assigned to a wait list group. The Research Team will ask the participants to complete various questionnaires over the course of this 6-month period, including at the beginning of the study. Before we conduct this study, we would like to figure out some of the reasons that will **help as well as prevent** people to participate in the study; stay in the study once they agree to participate in the study; and follow the study plan and Program. We also want to find out what you think are the important impacts/outcomes of the Peer Support Program.*

I. Experiences with/Expectations of OBIA Peer Support Program/Peer Support

Question	Stakeholder Group
1. **Tell me about your experience with the OBIA Peer Support Program**<br><br>*Probe: Why did you decide to join the OBIA Peer support program? What were your expectations/what were you hoping to get from the program/what did you think the program would offer you?*	Partners<br>Mentors<br>Caregivers<br><br>**who have** participated in the OBIA Peer Support Program
2. **Have you had any previous experience with a peer support program? If yes, tell me about that experience.**<br><br>*Probe: What were your expectations going into that peer support program? Or, if you have no previous experience of peer support, what are your expectations of peer support?*<br><br>3. **Before my description today, what had you heard about OBIA Peer Support Program?**	Partners<br>Mentors<br>Caregivers<br><br>who **have not** participated in the OBIA Peer Support Program
4. **From your perspective, what are the most important elements of the OBIA Peer Support Program or Peer Support in general?**	OBIA Staff<br>Researchers

II. Impact of OBIA Peer Support Program/Peer Support

Question	Stakeholder Group
5. **What was the impact of participating in the OBIA Peer Support Program?** *Probes: Impact on knowledge? Impact on mood? Impact on quality of life? Impact on confidence?*<br><br>*Probes:*<br>*a) Did it lead to any positive or negative outcomes for you?*<br>*b) What might be the best example that highlights the importance of peer support?*	Partners<br>Mentors<br>Caregivers<br><br>**who have** participated in the OBIA Peer Support Program
6. **What do you expect will be the impact of participating in the OBIA Peer Support Program (or another peer support program)?** *Probes: Impact on knowledge? Impact on mood? Impact on quality of life? Impact on confidence?*<br><br>*Probe: Do you anticipate any positive or negative outcomes of participating in the OBIA Peer Support Program (or another peer support program) and what are they?*	Partners<br>Mentors<br>Caregivers<br><br>who **have not** participated in the OBIA Peer Support Program
7. **From your perspective, what are the important outcomes of participating in the OBIA Peer Support Program or Peer Support in general, and why?**<br><br>8. **What are your thoughts on the following outcomes that we plan to examine?**<br>- **Participation**<br>- **Mood**<br>- **Health related quality of life**<br>- **Self-efficacy**	OBIA Staff<br>Researchers

III. **Enablers and Challenges to Participating in the Trial**

As mentioned, before we conduct this study, we would like to figure out some of the reasons that will ***help as well as prevent*** *people to participate in the study; stay in the study once they agree to participate in the study; and follow the study plan and Program.*

Question	Stakeholder Group
**8. How can we encourage people with brain injury to take part in this study? Can you think of any factors that might prevent people with brain injury from taking part in the study? Do you think any of these factors would be different for Partner versus Mentor participation? [recruitment]**<br>*Probe: From your perspective, what would help you/ participants decide to participate in this study?*<br><br>*Examples: where we advertise the study, the messaging around the importance of the study, potential impact of the study, compensation*	Partners<br>Mentors<br>Caregivers<br>OBIA Staff<br>Researchers
**9. Once people with brain injury join the study, what can we do to make sure that they will remain in the study? Can you think of any factors that might prevent people with brain injury from remaining in the study? Do you think any of these factors would be different for Partner versus Mentor participation? [retention]**<br>*Probe: From your perspective, what would help encourage you/ participants to stay in this study?*<br><br>*Examples: appropriate incentives, appropriate time commitment (e.g., program itself but also the outcome measures that are administered)*	Partners<br>Mentors<br>Caregivers<br>OBIA Staff<br>Researchers
**10. Once people with brain injury join the study, what can we do to make sure they follow the study plan and Program? Can you think of any factors that might prevent people with brain from following the study plan and Program? Do you think any of these factors would be different for Partner versus Mentor participation? [adherence]**<br><br>*Probes: For example, what reasons may help or prevent people from completing their questionnaires throughout the course of the 6 month study? Completing the sessions? Relationship with Mentor?*	Partners<br>Mentors<br>Caregivers<br>OBIA Staff<br>Researchers
**11. Overall, what plans can we put in place to make sure the study runs well? What factors might prevent these from happening?**	Partners<br>Mentors<br>Caregivers<br>OBIA Staff<br>Researchers

IV. Overall/Close of Interview

Question	Stakeholder Group
**12. Is there anything else you would like to add to our interview on your experiences with/expectations of the OBIA Peer Support Program? The impact of peer support on your life or what you expect it to have an impact on? How we can make this study run well for our participants/how we can minimize any challenges?**	Partners<br>Mentors<br>Caregivers
**13. Is there anything else you would like to add to our interview about the impacts or outcomes of the OBIA Peer Support Program or Peer Support in general? How we measure outcomes? How we can make this study run well for our participants/how we can minimize any challenges?**	OBIA Staff<br>Researchers
