# Supplementary material for: Using an integrated knowledge translation approach to inform a pilot feasibility randomized controlled trial on peer support for individuals with traumatic brain injury: A qualitative descriptive study
Source: PLoS One. 2021 Aug 24;16(8):e0256650. doi: 10.1371/journal.pone.0256650 (PMC8384186; doi:10.1371/journal.pone.0256650)
Supplement: S2 File — (PDF) [file pone.0256650.s002.pdf]

Hello Peer Support Coordinators,

We have received approval to begin recruiting for our study on the OBIA Peer Support Program!

As a reminder, our study is called “A Randomized Controlled Feasibility Trial on the Ontario Brain Injury Association Peer Support Program” – or “TOPS” for short. We are doing this study to look at what effects participating in peer support has on partners.

How long is the study and what kind of study is it?

This study will use a randomized controlled method and will last 4 months. This means that when partners enroll in the study, they will be assigned by chance into one of two groups:

1) Receiving peer support
2) Wait-list control (this means the partner won’t be matched with a mentor until after 4 months)

What do partners and mentors in the study need to do?

- Anyone interested in being part of the study will have to go through a screening process by phone with the research team and provide their informed consent to participate
- Over the 4 months of the study
    - Partners will either be getting peer support or waiting for peer support, and will do surveys by phone with the researchers at 3 different times
    - Mentors will do surveys by phone with researchers at 3 different times, fill out their logs as usual, and they might be asked to meet with researchers by phone

What do people get for being in the study?

People will receiving an honorarium of a $100 gift card for participating in the study

What we need your help with:

Informing your mentors about the study

Please let your mentors know that this study is happening and that they may be asked to be involved if they are matched with a partner who is in the study. We have an information letter that we’ve prepared that you can send (*see document titled: “Study information flyer for mentors pre-recruitment”*). They do not need to contact researchers at this point. If they’re partnered with someone in the study, they’ll get an invitation to be part of the study and be asked to contact researchers then.

Recruiting partners for the study

As you do your intake of new partners (or if you have partners on your list that haven’t been matched yet), we ask that you tell them about the study/pass on the recruitment flyer (*see document titled: Recruitment Flyer partners*).

We are looking for Partners who

- have not previously participated in the OBIA Peer Support Program
- are not currently receiving any other type of peer support
- have moderate to severe traumatic brain injury
- 18 years or older
- fluent in English

You don't need to figure out 100% whether someone is eligible, as some cases might be tricky. This is just to give you a general idea. If you know for sure someone would not be eligible, no need to tell them about the study – but if you think someone might qualify, even if you're not sure, please pass on the information!

If the partner is definitely not interested – there's no need for them to contact us. Continue with your intake and matching process as normal.

If the partner is potentially interested, you can let them know to contact me (Dorothy). If you want to email/call to give me a heads up, that would be great – that way I can stay in contact with you about whether this person has enrolled (and whether they'll be in the group that receives peer support right away vs the wait list) or whether they won't be in the study and that you should continue your intake as usual.

<u>Matching partners who are in the study</u>

Only partners in the study who are in the peer support group should be matched with a mentor right away. You would find mentors for partners in this study the same way you normally do. Once you have a suitable mentor for the partner, please pass on the recruitment flyer (*see document titled: Recruitment Flyer mentors)*/ask that the mentor contact Dorothy. This will be their invitation to enroll in the study.

<u>How long we need your help for:</u>

We are looking to get 40 Partners into the study (and 20 mentors). We'll recruit until we reach that number, or until the end of May 2019.

<u>Thank you!</u>

We'd like to thank you all in advance for your help in making this study happen. I know there are a lot of details about the study and eligibility can be tricky to figure out, so please don't feel like you need to have all the answers – that's what I'm here for! Don't hesitate to let people know that they can contact me directly to chat about the study, and don't hesitate to contact me yourselves if you have questions. I look forward to touching base with you all throughout the next several months. Thank you!

Dorothy Luong
