# Supplementary material for: Using an integrated knowledge translation approach to inform a pilot feasibility randomized controlled trial on peer support for individuals with traumatic brain injury: A qualitative descriptive study
Source: PLoS One. 2021 Aug 24;16(8):e0256650. doi: 10.1371/journal.pone.0256650 (PMC8384186; doi:10.1371/journal.pone.0256650)
Supplement: Attachment — Submitted filename: Response to Reviewers.pdf [file pone.0256650.s004.pdf]

Response to Reviewers

Dear Editor:

Thank you for your email. I am pleased to hear that you believe that our manuscript will have the potential to be published in PLOS ONE following requested revisions.

As per your request, please find our response to the peer-reviewers' comments with every change outlined point by point below:

==

Editor and Reviewer comments:

Reviewer #1: The aim of this article is two explore key informants'perspectives (iKT) of barriers and facilitators of participating in peer support interventions and of its effectiveness, among people with TBI. Finally, the authors try to demonstrate how iKT can be useful in the development of a RCT.

The article is well-written in a correct English.

TBI affects millions of people each year worldwide. In addition to the physical and cognitive deficits, TBI can lead to depression, anxiety and reduced quality of life. A promising and cost-effective interventions is peer support.

The topic of the article is interesting and not extensively development yet in neurosurgery. However, the article is, currently, too long and not completely focused on the aim of the study. The article is interesting but it is too long, this means that it is difficult to keep the attention throughout the reading. I think that the paper should be shorter and more tailored on the aim of the study.

- We thank the reviewer for taking the time to provide their feedback on our manuscript. We have taken this feedback into consideration when shortening our manuscript to being more focused on the objectives.

Reviewer #2: Integrating peer support into rehabilitation of severe TBI'S patients is an interesting topic.
However, multicenter studies are needed to validate utility in different countries.
Furthermore this study shows the useful integration of iKT in the construction of the RCT protocol.
the work is well written and I thank the authors for their submission.

- We thank the reviewer for taking the time to provide their feedback on our manuscript.

Academic Editor: Please revise the article according the Reviewers' comments. Reduce the lenght of the manuscript to improve its readability.

- We thank the editor for taking the time to provide their feedback on our manuscript. We have reduced the length of the manuscript as suggested.

==

- Again, we thank the reviewers and editor for providing their feedback on our manuscript and we have taken all their feedback into consideration when revising our manuscript. Additionally, as

per the journal requirements, our manuscript meets PLOS ONE’s style requirements, we specified the participant consent process, we elaborated on our methods section, and we reviewed our reference list to ensure both completeness and correctness. However, in regard to the minimal underlying data set, the relevant data are within the manuscript/supporting information files, and we cannot share the full transcripts publicly as the participants did not consent to such. However, researchers can request for access to these transcripts via the University Health Network’s Research Ethics Board.

===

Should your editorial office require any further edits following my most recently submitted submission, please do not hesitate to inform me and we will make these changes as soon as possible. Thank you for your consideration.

Yours sincerely,

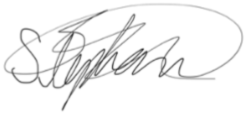

Stephanie KC Lau, BSc, MD Candidate, on behalf of the study authors
SLau065@uottawa.ca
